# Surface Orientation and Binding Strength Modulate Shape of FtsZ on Lipid Surfaces

**DOI:** 10.3390/ijms20102545

**Published:** 2019-05-24

**Authors:** Ileana Márquez, Gabriel Díaz-Haro, Marisela Vélez

**Affiliations:** Instituto de Catálisis y Petroleoquímica, CSIC. c/ Marie Curie, 2, Cantoblanco, 28049 Madrid, Spain; ile.f.marquez@gmail.com (I.M.); gabriel.diaz.haro@gmail.com (G.D.-H.)

**Keywords:** FtsZ, bacterial cytoskeleton, atomic force microscopy, lipid membranes, filament torsion

## Abstract

We have used a simple model system to test the prediction that surface attachment strength of filaments presenting a torsion would affect their shape and properties. FtsZ from *E. coli* containing one cysteine in position 2 was covalently attached to a lipid bilayer containing maleimide lipids either in their head group (to simulate tight attachment) or at the end of a polyethylene glycol molecule attached to the head group (to simulate loose binding). We found that filaments tightly attached grew straight, growing from both ends, until they formed a two-dimensional lattice. Further monomer additions to their sides generated a dense layer of oriented filaments that fully covered the lipid membrane. After this point the surface became unstable and the bilayer detached from the surface. Filaments with a loose binding were initially curved and later evolved into straight thicker bundles that destabilized the membrane after reaching a certain surface density. Previously described theoretical models of FtsZ filament assembly on surfaces that include lateral interactions, spontaneous curvature, torsion, anchoring to the membrane, relative geometry of the surface and the filament ‘living-polymer’ condition in the presence of guanosine triphosphate (GTP) can offer some clues about the driving forces inducing these filament rearrangements.

## 1. Introduction

FtsZ is a bacterial cytoskeletal protein that binds and hydrolyzes GTP to self-aggregate into dynamic filaments and guide the assembly of the septal ring on the inner side of the membrane at midcell that constricts the cell during division. FtsZ is conserved throughout bacteria, both gram-positive and gram-negative, archaea [1] and exists in chloroplasts and in the mitochondria of certain eukaryotes [2].

Ever since its discovery near 25 years ago, its great polymorphism and dynamic behavior have raised many questions regarding how such a dynamic and polymorphic structure can be responsible for force generation [3,4].

The structures of FtsZ monomers of different organisms have been determined. They are known to assemble head-to-tail forming polar filaments [5,6,7]. FtsZ consists of an N-terminal GTP binding domain and a C-terminal GTPase-activating domain linked by the long core helix H7 and loop T7. Upon filament formation, the C-domain from the upper subunit contacts the N-domain and the top of H7 of the lower subunit to complete the GTPase site [8,9]. This engages cooperative polymerization and GTP binding/hydrolysis, which requires the fine coupling of both domains, probably by combination of elements including hinges, moving parts, and energetically coupled residue networks providing signal transmission across monomers [10].

Recent computational studies have shown that the monomers flexibility is important and can be involved in the apparent treadmilling behavior [11]. Another important feature of the FtsZ monomers is the presence of a structural flexible region, the CTL (carboxi-terminal linker) [12]. FtsZ is tethered to the membrane by binding, with moderate affinity [13,14], to membrane-associated proteins ZipA and FtsA through a C-terminal conserved region [15,16]. The CTL spans the gap between the main protein core and this conserved terminal region, suggesting that a flexible connection between FtsZ protofilaments and the membrane is required. ZipA also has a flexible linker between the C-terminal FtsZ-interacting domain and its transmembrane region [17], a fact that reinforces the idea that a malleable and adaptable association of FtsZ to the membrane is important to modulate interactions both between its subunits and between other modulatory proteins in the cytoplasm [18].

Until now, most of the information regarding the importance of the flexible linkage to the membrane comes from genetics, biochemistry and cytology studies [12]. This region, present in all FtsZ although with different lengths, has been shown to play an important role in vivo [18], modulating interactions between subunits, their interaction with the membrane, and affecting peptidoglycan synthesis [19]. Some of this work has been done in cells, using high-resolution fluorescence techniques, which do not provide enough resolution to propose a mechanism. It is, therefore, still difficult to understand how this flexible region that lies between the filaments and the surface affects and governs their structure and function. 

In vitro studies in solution have tried to address the question about how this CTL affects filament structure and dynamics. One conclusion is that the disordered C-terminal tails participates into the lateral association of the filaments to form bundles [6,18,20]. However, experiments in solution do not reproduce in vivo conditions in which filaments are oriented on the inner membrane surface. In recent years, experiments in reconstituted model systems have shown that the assembly on lipid surfaces in model membranes has a strong effect on the structure and dynamics of the filaments [14,21,22]. Orientation and constriction to a two-dimensional surface seem to unleash a spatially coordinated collective dynamic behavior [23,24] previously unexpected. Recent results indicate that the CTL also affects this dynamic behavior observed on surfaces [25].

Whether these complex dynamic surfaces formed on planar two-dimensional surfaces resemble what happens inside the cells during division is still to be determined. However, it does indicate that orienting filaments on a surface provides additional elements to reveal an even wider polymorphism and dynamic behavior. It could be that the non-structured region lying between the filaments and the membrane could play a role. Strategies to artificially attach and orient FtsZ to membranes have provided useful information about structural and dynamic properties of the filaments [26,27,28]. Even if these results obtained in model systems cannot be directly extrapolated to the more complex behavior in vivo in the presence of other modulating proteins, they have been extremely useful to reveal intrinsic properties of the filaments. The sensitivity to the lipid composition of the membrane [21], capacity to exert force and deform an underlying membrane [27,28], and the impact of the monomer orientation on the surface [22] observed in vitro are likely to play a role also in the functional behavior of FtsZ in vivo.

Theoretical studies describe the rich behavior of filaments on a two dimensional surface [4,29,30,31,32,33,34,35]. The elements included in the theory to explain the experimental results include the presence of a preferential curvature, lateral interactions and a twist between monomers. Although a twist had also been suggested previously from in vitro studies [11,36] and MD simulations [37] its impact on the structure and mechanical properties of filament aggregates was not explored. The scenario offered by this theoretical model predicts that the strength and orientation of filament attachment to the surface modulates the twist and, consequently, the shape and rigidity of the filaments on the membrane.

In this study we seek to test this hypothesis. We have used the same strategy described previously [22] to covalently anchor a mutant form of *E. coli* FtsZ containing a cysteine in the amino terminal domain F2C-*Ec*FtsZ to a lipid membrane. We have used two different lipids to anchor the protein, one containing the maleimide anchoring motif located in the phospholipid polar head, to simulate a tight binding, and one containing the maleimide anchoring motif at the end of a PEG spacer bound to the polar lipid head. This artificial covalent attachment takes place through the amino terminal domain of the protein, where the cysteine mutation is located. This orientation is approximately 180 degrees from the C-terminal domain through which the proteins are attached in vivo. However, the results serve to illustrate the impact of the surface attachment on FtsZ filament behavior and can shed some light on the potential function of being attached to the membrane through a flexible linker.

## 2. Results 

**Figure 1:**
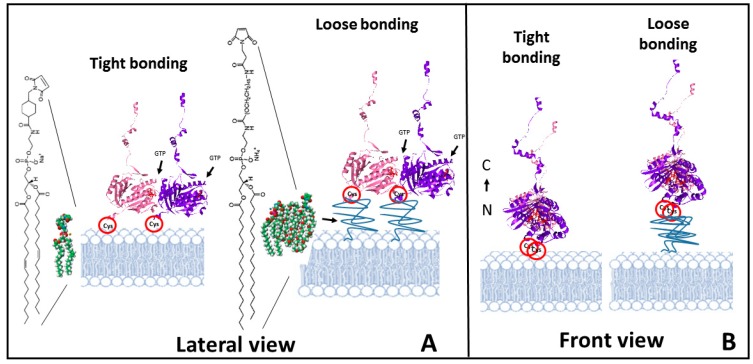
Schematic illustration of the experimental approach. (**A**) shows the lateral view of a dimer of F2C-*Ec*FtsZ attached to a lipid bilayer through the cysteines. The type of maleimide containing lipids introduced at 5–10% in the lipid membrane used to simulate either a tight binding (left) or a loose binding (right) are shown. (**B**) Front view of the filaments to illustrate the orientation of the monomer and the availability of lateral sites for interaction. The orientation of the N terminus -C terminus axis of the monomers is shown.

The strategy used to orient proteins on a lipid membrane is based on the introduction of a cysteine group within the protein that can be covalently anchored to a maleimide containing lipid included in the planar lipid bilayer. *Ec*FtsZ has no cysteines [38], so introducing such an amino acid places a reactive site at a known location on the protein surface. The cysteine mutant used is F2C-*Ec*FtsZ, in which the phenylalanine in position 2 is substituted by a cysteine. The mutation is located outside the monomer−monomer interface region involved in the protofilament formation [39] and away from the polymerization direction. Therefore, both polymerization and GTP hydrolysis are possible, even with the protein oriented facing the surface. The mutant preparation and characterization are as described in [22]. The use of maleimide−poly(ethylene glycol)-derivatized phospholipids to bind proteins through sulfur-containing cysteines to liposomes has been previously described [40,41]. We adapted this strategy to attach the monomer form of the self-assembling bacterial cytoskeletal protein directly to a planar lipid bilayer. We used two types of maleimide lipids: one, with the maleimide anchoring group on the lipid polar head, with no poly (ethylene glycol) (PEG) spacer, to impose strong orientational restrictions on individual monomers to simulate a tight binding. The other lipid used contains the maleimide moiety at the end of a PEG spacer (2000 MW), to simulate a looser binding (Figure 1). 

### 2.1. Tight Binding

**Figure 2:**
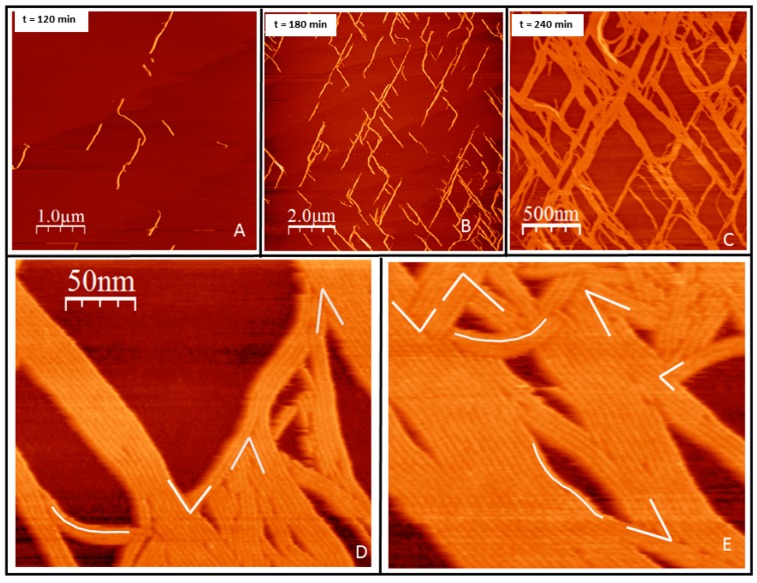
Filament growth of tightly bound protein in two dimensions. (**A**) initiation of filament growth in two dimensions two hours after GTP addition. (**B**) Sixty minutes later filaments have elongated in two preferential directions at approximately 60 degrees of each other. (**C**) One hour later, lateral addition of monomers has given rise to filament bundles. Time indicates minutes after GTP addition. (**D**,**E**) Zoom of two area shown in C to illustrate the diversity of angles and curvatures. Most of the angles are contained in a range between 85 and 35 degrees. White lines guide the eye to illustrate the range of angles and curvatures observed.

**Growth in two dimensions.** F2C-*Ec*FtsZ was incubated for over one hour on a supported lipid bilayer composed of DOPC lipids containing 5–10% molar fraction of PE MCC-MAL, to assure complete coverage of the lipid surface by covalently attached protein. GTP was added at 5 mM concentration, after rinsing the excess protein remaining in the solution. Figure 2 shows the linear structures formed on the surface after GTP addition. Filaments start nucleating visibly on the surface after approximately one hour after nucleotide addition. The structures formed are straight and highly oriented (Figure 2). The growth initiates independently at different positions and takes place by addition of monomers at the ends (Figure 2A). It is remarkable that there are mainly two orientations, although the separation between nucleation sites is of hundreds of nanometers, orders of magnitude more than the thickness of the individual filaments. Growth occurs at both ends until the presence of other filaments stops it. Ends become less available and the mesh then grows by addition of monomers on the sides of existing filaments (Figure 2B). From the AFM images, we cannot distinguish the type of interaction between monomers, but can only assume that they are similar to the ones previously described, longitudinal in the presence of GTP and lateral due to lateral monomer-monomer interactions [4]. Steric hindrance prevents overlap between adjacent monomers during the initial stages of filament formation and lateral bundling.

The mainly straight bundles formed by the lateral interactions retain certain local flexibility. They do not cycle as they do on mica [42] but they undulate to different extents. Filaments observed at the micrometer range grow in two preferential orientations aligned at 60 degrees, following the expected orientation of the mica (Figure S1). When observed with nanometer resolutions, the contact between bundles occurs at several possible angles (Figure 2D,E). We interpret this data as indicating that the underlying lipids, probably due to the tension generated by the filaments, guide their orientation to follow that of the mica surface. 

**Figure 3:**
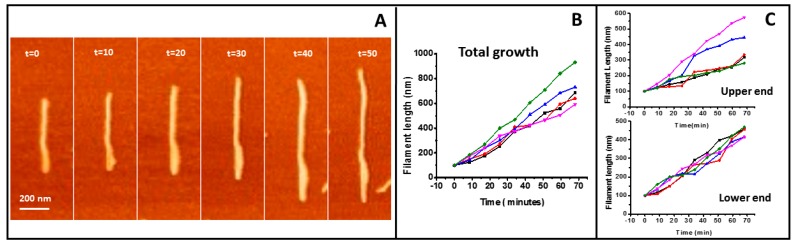
Filament growth speed of tightly bound protein. (**A**) illustrates the growth of one individual filament (time indicated in minutes). (**B**) shows graphs of the total growth rate of five independent filaments. The graphs were shifted vertically to normalize for length differences between filaments. (**C**) shows the growth at the two ends of the filaments.

Figure 3 shows the quantitative analysis of filament growth. Filaments a few tens of nanometers long and around 20 nm in width can be easily identified. As the width of isolated structures is limited by the tip diameter, typically 10–20 nm, we assume these are single filaments. We do not have enough spatial and time resolution to detect the addition of individual monomers, but growth due to the incorporation of a few of them to each end can be detected and quantified. The total growth speed of five filaments is shown in Figure 3B. We also analyzed the growth from the independent ends (Figure 3C) (SMov1 in Supplementary Material). The mean average of the 5 filaments presented is 9.2 ± 1.9 nm/min for the total growth and 5.0 ± 0.6 nm/min for one end and 4.3 ± 2 nm/min for the other end. We do not observe any significant difference in the growth rate of the individual filament ends.

**Figure 4:**
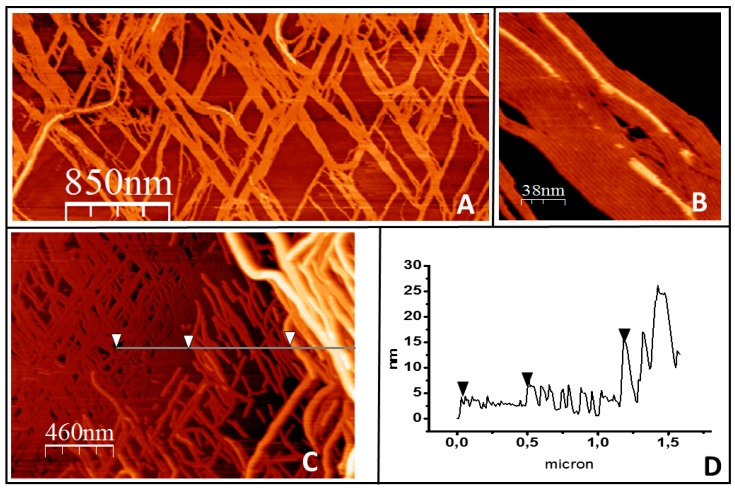
Filament growth of tightly bound protein in three dimensions. (**A**) shows a general overview of filament bundles on the lipid surface. (**B**) shows how the filaments growing on top closely follow the orientation of the filaments below. (**C**) illustrates that in the areas where the lipids become fully covered by the protein, the second protein layer develops into ever higher and more complex structures. The height profile of the line is represented in (**D**). The arrows in both panels are a guide to show the three height levels observed: the first layer of filaments, the second layer, and a third layer of thicker structures.

**Growth in three dimensions.** After several hours of observation, filaments start growing away from the mica surface, in spite of the fact that, in principle, the only proteins available are linked covalently to the lipids (Figure 4). A second layer of protein grows at some places strongly associated with the first layer formed directly on top of the lipids. Figure 4A shows a low-resolution image in which a second layer, observed as lighter colored filaments, appear at some points. Figure 4B shows a close-up view that illustrates that growth closely follows the filament lying underneath. The thickness corresponds to one protein layer. Figure 4C shows that, once a first layer of protein fully covers the lipid surface, the formation of a second layer extends. At certain locations, this three-dimensional mesh forms bundles that are well above the thickness of a pure protein layer. Figure 4C shows that these aggregates can be as 25 nm at some points. The bilayer becomes unstable after these aggregates appear, and it is not possible to follow their evolution. The bilayer ends up detaching from the surface, possibly due to the tension generated by the protein rearrangement.

### 2.2. Loose Binding

**Figure 5:**
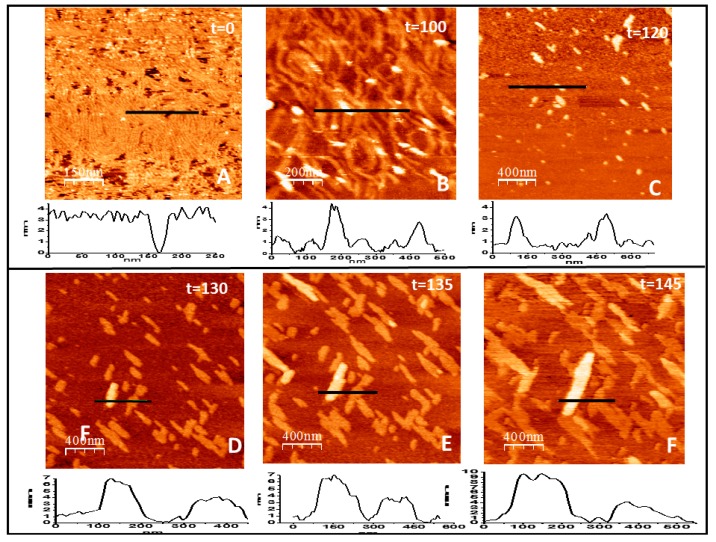
Filament growth of loosely bound protein in two and three dimensions. (**A**–**F**) show the time evolution (in minutes) of the structures formed by FtsZ attached to the lipid surface through a flexible PEG linker immediately after GTP addition (*t* = 0). The graph below each image represents the topographical profile acquired at the position indicated by the horizontal line (all numbers are in nm). Well defined individual filaments formed immediately after GTP addition (**A**) condense into thicker curved bundles (**B**) that later disaggregate, leaving only some agglomerates that retain the height of 4 nm of the filaments (**C**). After a few minutes, condensation takes place again (**D**) and leads to the formation of linear arrangements that recover the height of one and two layers of lateral filament bundles that grow both longitudinally and laterally at a defined orientation (**E**,**F**).

The same experiments were performed using the lipid PEG derivative present at 5% in the lipid to obtain full protein coverage of the surface. Figure 5A shows the type of structures formed immediately after the addition of GTP. Filaments adopt the same type of flexible curved filaments observed on mica at high protein densities [42]. The structures are unstable and evolve in time, as expected in experiments performed in the presence of GTP. Individual filaments become less defined and condense into thicker bundles (Figure 5B). Surprisingly, after two hours after GTP addition, defined filaments disappeared (Figure 5C). A few minutes later the protein condenses again into linear bundles aligned in two preferential directions, as happened in the tightly bound protein described above. In this case, longitudinal and lateral growth are not clearly distinguishable and the bundles growth in both directions in a more coordinated way (Figure 5D–F). The fixed orientation of the monomers to the lipids seems to override the initial flexibility of the binding through the PEG molecule. We also observe that a protein layer, one monomer thick, forms on top of the bundles attached to the lipids. After the protein layer becomes dense and fully covers the lipids, the structures become unstable and the lipid and protein layers detach from the mica, preventing further observation. 

The flexible linker gives the proteins degrees of freedom to organize first as individual curved filaments and later condense into lateral bundles. This second stage condensation shares some traits with the filaments formed through a tight binding. In both cases, the filament bundles follow two preferred orientations at 60 degrees of each other, but in this case the growth of individual filaments cannot be observed. In both cases, we observe at later times the formation of a second layer of filaments that precedes membrane rupture. 

## 3. Discussion

Results presented illustrate the complexity of the polymerization behavior of FtsZ on surfaces. We observe clear differences in FtsZ polymerizing response depending on the binding strength. The first significant difference is the time response to the addition of GTP. Whereas FtsZ bound through a flexible linker to the surface responds immediately forming filaments, the tightly bound protein presents a lag time of hours before the first filaments appear. In the case of the tight bonding, the GTP pocket lies close to the lipid bilayer and diffusion of the charged GTP molecule into this hidden pocket is likely to be sterically hindered (Figure 1). The loose bond through the PEG-lipid permits access of the GTP to the monomers on the surface allowing immediate filament formation. 

The expectation was, according to the hypothesis that filaments in solution show a certain torsion [33], that tight anchoring of the monomers to the surface through the amino terminal region would promote its cancelation, whereas loose bonding would not. Filaments oriented with their preferential curvature lying perpendicular to the lipid membrane and a tight binding would adopt a straight configuration when seen from above [4,33]. This is indeed what happened in the initial stage of filament formation. Figure 2 illustrates that upon tight binding, filaments are mostly straight, whereas when bound loosely, monomer torsion is not restricted and filaments can adopt curved conformations, similar to the ones found on mica at high surface protein densities [42] (Figure 5A). Tightly bound filaments, however, retain certain flexibility. They do not close into rings, as they do on mica or on lipid bilayers when attached through an artificial membrane anchor [23,24], but they retain the capacity to undulate (Figure 2D,E). The bond to the membrane still allows for certain twisting that could be responsible for rendering this additional flexibility [33]. Rings on the surface should only be present if the anchoring is loose enough to allow the plane of the main curvature to lie parallel to the lipid surface. As this plane lies along the axis between the amino and carboxy terminal domains [33], only certain surface attachments can allow this. 

Experiments were performed in the presence of GTP with a fixed amount of protein covalently attached to the surface. We, therefore, expected to observe filaments evolve in time, due to their well-known “living polymer” character [42,43], that allows monomer exchange, fragmenting and annealing in the presence of GTP. However, the big differences in the time evolution of the filaments observed in the two types of attachments were not expected. The tightly bound filaments did not show any breaking or reannealing, only progressive longitudinal and lateral growth, whereas the filaments bound loosely underwent dramatic transformations. After an initial reorganization, including filament breaking and monomer reshuffling, aggregates showing stable growth and similar in shape and orientation to the ones observed in the tight binding appeared (Figure 5). This observation indicates that the complex dynamic behavior of the filaments is closely related to how filaments are associated with each other and to the surrounding surface. The dynamic stability of the tightly bound filaments could indicate that the inaccessibility of the GTP pocket hampers nucleotide hydrolysis and exchange. 

The results presented also highlight the importance of lateral interactions in guiding filament bundling. The structural information obtained from tightly bound protein allowed visualizing the range of contact angles found in intercepting filaments (Figure 2D,E). It is clear that these lateral interactions are not strongly restricted, and filaments can associate forming many different angles. Much has been said about the importance of lateral interactions in the bundling and functioning of FtsZ filaments, but establishing the exact contact regions and their nature has remained elusive [14,44,45,46,47]. Recent crystallographic data and in vivo studies [48] have confirmed that lateral interactions are relevant for function and suggest they are predominantly mediated by van der Waals interactions, making them sensitive to surface geometry. Those results also suggest that lateral interactions between FtsZ protofilaments are weaker on a per subunit basis in comparison with hydrophobic longitudinal interactions, making protofilament preassembly a requirement for lateral interactions to occur. This is exactly what we observe in the case of the filaments formed from proteins tightly bound to the lipids: they grow mostly in longitudinal and lateral interactions are only prevalent after filaments form. 

Filaments formed from loosely bound proteins follow a different route to condense into oriented bundles (Figure 5D–F). At initial stages, the twist provides the flexibility required for the formation of undulated filaments, as expected. Later, the spatial restriction imposed by protein attachment limits further two-dimensional reorganizations. The oriented protein proximity favors formation of lateral interactions between monomers that can stabilize or lock certain monomer conformations similar to the ones achieved during the tight bonding. In this case, however, lateral and longitudinal interactions become less independent to guide bundle assembly. Unfortunately, the resolution of the images obtained is not as good as the one obtained for strongly attached filaments. The PEG present between the filaments and the lipid bilayer prevents the sample stiffness convenient for optimal imaging.

**Figure 6:**
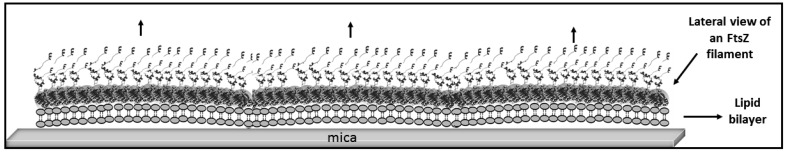
Schematic illustration of FtsZ filament stiffening by twist cancellation. The cartoon depicts the possible filament arrangement on the membrane after twist cancellation that leaves the preferential curvature oriented downward lying on a plane perpendicular to the membrane. The arrows indicate the direction of the force that could contribute to destabilize the membrane.

Growth in three dimensions is another unexpected observation. Proteins are covalently attached to the lipids, so it is not obvious to understand how they become available to form an additional filament layer away from the lipid surface. One possibility is that the high local cysteine density allows for certain extent of thiol substitution [49], releasing some FtsZ monomers. This could explain the origin of the protein forming a second layer of filaments. An outstanding feature is that they closely follow the shape of the filament below and that their growth extends at localized regions once it´s initiated (see Figure 4B). Lateral interactions are likely responsible for this. The carboxy terminal region is facing upwards (see Figure 1B), so a second layer can only form through oriented lateral interactions between monomers in the upper and lower layers. Once this happens, the upper layer will impose restrictions to the twist of the lower filament that could propagate along the longitudinal axis facilitating further addition of monomers above with the same orientation. This is consistent with the proposed surface geometry sensitivity of lateral interactions mentioned above. Once a dense layer of filaments covers the lipid surface, we observe that the second filament layer leads to the formation of even higher structures that are not structurally so well defined (Figure 4C and Figure S2). Their thickness, height, and longitudinal appearance indicate that they are formed by proteins and lipids pulled out of the membrane. Lipids amphipathic character would contribute to the self-assembly of these larger structures that could represent the initial stages of the bilayer disruption observed at longer times. One possibility is that the increasing number of lateral interactions impose restrictions on the overall filament structure, both on twist and orientation, locking monomers in a certain position. Information provided by molecular modeling indicates that the energy to untwist the filament is smaller than the energy required to unbend the preferential curvature [33]. Protein orientation forced by the covalent bond to the lipid, with the *N*-Carboxy-terminal axis lying perpendicular to the surface, predicts that filaments will curve downwards [28]. If the twist that softens the filaments is cancelled, filament curvature perpendicular to the plane of the membrane could exert force on the lipid membrane (Figure 6).

This same argument can be used to explain another unexpected observation. Figure 2 and Figure S1 show that the filaments follow the orientation of the mica surface, in spite of the fact that the lipid bilayer lies between them. This orientation is also observed on the second stage rearrangement of the loosely bound filaments (Figure 5D–F). The tension on the underlying membrane generated by the orientation of the filaments could couple the orientation of the lipids to the mica surface. It is then likely that membrane tension orients filaments that are nucleating at a large distance between them. The fact that this effect is also observed on the proteins attached through a PEG linker confirms that this polymer is providing flexibility but not separating the proteins from the surface. Figure S3 shows that the distance between the proteins and the lipid bilayer is not significantly different between the two types of protein anchoring. Previous studies have reported that the presence of PEG does not affect the distance between a bilayer and a solid surface [50]. It is well known that mica can have an ordering effect on peptides [51], but to our knowledge, it is the first time that the ordering is seen to be transmitted across the lipid bilayer to affect protein growth on the surface. 

One important result is that we do not observe treadmilling during individual filament growth. Experiments both in vivo [52,53] and in vitro have described filament treadmilling, that is, preferential growth at one filament end and loss at the other [23,24]. Under our conditions, both filament ends grow with no significant speed difference (see Figure 3). The polymerization-associated conformational switch detected by high-resolution structural analysis of filaments [11] was proposed to explain treadmilling of single-stranded filaments, but it cannot account for our observations. We have previously argued that, if both twist and curvature are considered, filament orientation on a membrane imposes geometric restrictions that could account for asymmetric growth [4]. Treadmilling would be observed for filaments not covalently attached to the membrane in which twist would induce different accessibilities of the end monomers, one positioned away from the membrane and the other buried towards it [4]. In the experiments presented here, the covalent attachment, particularly the tight one, for which individual filament growth is clearly observed, the twist responsible for this asymmetry is canceled, leaving end monomers equally available for polymerization. The preferential filament curvature facing perpendicularly downward towards the membrane could also contribute to cancel the twist effect and promote symmetric end growth.

Experiments presented here show that protein attachment and orientation have a strong impact on filament shape, growth and bundling, as predicted by a filament model that considers filament torsion. Although these experiments do not directly measure mechanical properties, our results suggest that the other prediction, that filament rigidity is affected by the attachment, is also indirectly detected. We see that filament orientation is coupled to the mica surface through the membrane and that membrane collapses after the protein fully covers the surface (Figure S2), compatible with an increased filament stiffness when twist is cancelled. This simple experimental setup consisting of protein and lipids on a surface highlights the rich landscape of complex structures available by the inbuilt design of the FtsZ bacterial protein. Lateral interactions, the balance between the spontaneous curvature and torsion, the anchoring to the membrane, the relative geometry of the surface and the filament and its ‘living-polymer’ condition in the presence of GTP-GDP all come into play and provide reasonable explanations to our experimental observations. It will be important to keep in mind the rich intrinsic complexity inbuilt in the FtsZ filaments when trying to understand their role in vivo and their regulation by the large set of proteins that participate in Z ring formation and function. 

## 4. Materials and Methods 

The production, overexpression, and purification of the F2C-*Ec*FtsZ were done as described previously [22].

**Preparation of F2C-*Ec*FtsZ Anchored to Planar Lipid Bilayers.** Separate 0.1 M stock solutions of the lipids dioleoyl phosphatidylcholine (DOPC, 786 Da, Avanti Polar Lipids), 1,2-dioleoyl-sn-glycero-3-phosphoethanolamine-*N*-[4-(p-maleimidomethyl)cyclohexane-carboxamide] (sodium salt) 18:1 PE MCC (Avanti Polar lipids) and 1,2-distearoyl-sn-glycero-3-phosphoethanolamine-*N*-(maleimide(polyethylene glycol)-2000) (ammonium salt) DSPE-PEG (2000) Maleimide ( Avanti Polar lipids) were prepared in CHCl_3_/CH_3_OH 1/1 (*v*/*v*) solvent. A mixture of 90% DOPC/10% PE MCC-MAL (mol/mol) or 90% DOPC/10% DSPE-PEG MAL, depending on the experiment, was evaporated under nitrogen and resuspended in buffer L (50 mM Tris-HCl (pH 7.4), 200 mM NaCl, 5 mM CaCl_2_) at a final lipid concentration of 2.5 mM. To obtain large unilamellar vesicles (LUVs), the suspension was extruded 31 times through a 200-nm-pore membrane. To fuse the lipid bilayer on the substrate, a dilute 0.1 mM solution of the LUVs was placed in contact with freshly cleaved mica for 45 min at 30 °C. Then, the samples were rinsed with buffer Z (50 mM Tris-HCl (pH 7.4), 500 mM KCl, 5 mM MgCl_2_) to remove excess LUVs. To anchor each FtsZ mutant to the lipid bilayer surface, a 2 μM solution of the protein in buffer Z was incubated on the formed bilayer for several hours to ensure complete coverage of the active surface, under conditions similar to those used to attach cysteine-containing proteins to maleimide-containing surfaces [54]. The amount of protein in a 2 μM solution is in large excess with respect to the amount of lipid linker head on the surface. Buffer Z is an appropriate environment for FtsZ, as the high ionic strength prevents self-association and magnesium and potassium are important ions for nucleotide-dependent polymerization and GTP hydrolysis [55]. In addition, 100 μM TCEP was added to reduce eventual disulfide bonds formed between proteins. After the incubation period, the sample was rinsed with buffer Z to remove excess protein. 

**Atomic Force Microscopy.** Atomic force microscopy (AFM) imaging was performed on the bilayer-anchored FtsZ mutants in buffer Z in the absence and presence of 5 mM GTP to study the polymerized state. AFM images were recorded with a microscope from Nanotec Electrόnica (Madrid, Spain) operated in jump mode [56] in a liquid environment and in an Agilent 5500 Scanning Atomic Force Microscope (Keysight Technologies, Santa Rosa, CA, USA) operated in tapping mode. The scanning piezo was calibrated using silicon calibrating gratings (NT-MDT, Moscow, Russia). Silicon nitride tips (Veeco) with a force constant of 0.05 N/m and a 20-nm tip radius were used.

## Supplementary Materials

Supplementary materials can be found at https://www.mdpi.com/1422-0067/20/10/2545/s1.

## References

[1] Wang X., Lutkenhaus J. (1996). Ftsz ring: The eubacterial division apparatus conserved in archaebacteria. Mol. Microbiol..

[2] Gilson P.R., Beech P.L. (2001). Cell division protein ftsz: Running rings around bacteria, chloroplasts and mitochondria. Res. Microbiol..

[3] Mingorance J., Rivas G., Vélez M., Gómez-Puertas P., Vicente M. (2010). Strong ftsz is with the force: Mechanisms to constrict bacteria. Trends Microbiol..

[4] Mateos-Gil P., Tarazona P., Vélez M. (2019). Bacterial cell division: Modeling ftsz assembly and force generation from single filament experimental data. FEMS Microbiol. Rev..

[5] Matsui T., Yamane J., Mogi N., Yamaguchi H., Takemoto H., Yao M., Tanaka I. (2012). Structural reorganization of the bacterial cell-division protein ftsz from staphylococcus aureus. Acta Crystall. Sect. D.

[6] Huecas S., Ramírez-Aportela E., Vergoñós A., Núñez-Ramírez R., Llorca O., Díaz J.F., Juan-Rodríguez D., Oliva M.A., Castellen P., Andreu J.M. (2017). Self-organization of ftsz polymers in solution reveals spacer role of the disordered c-terminal tail. Biophys. J..

[7] Nogales E., Wolf S.G., Downing K.H. (1998). Structure of the αβ tubulin dimer by electron crystallography. Nature.

[8] Oliva M.A., Cordell S.C., Lowe J. (2004). Structural insights into ftsz protofilament formation. Nat. Struct. Mol. Biol..

[9] Scheffers D.J., de Wit J.G., den Blaauwen T., Driessen A.J. (2002). Gtp hydrolysis of cell division protein ftsz: Evidence that the active site is formed by the association of monomers. Biochemistry.

[10] Martín-Galiano A.J., Buey R.M., Cabezas M., Andreu J.M. (2010). Mapping flexibility and the assembly switch of cell division protein ftsz by computational and mutational approaches. J. Biol. Chem..

[11] Wagstaff J.M., Tsim M., Oliva M.A., García-Sanchez A., Kureisaite-Ciziene D., Andreu J.M., Löwe J. (2017). A polymerization-associated structural switch in ftsz that enables treadmilling of model filaments. mBio.

[12] Buske P.J., Levin P.A. (2013). A flexible c-terminal linker is required for proper ftsz assembly in vitro and cytokinetic ring formation in vivo. Mol. Microbiol..

[13] Hernández-Rocamora V.M., Reija B., García C., Natale P., Alfonso C., Minton A.P., Zorrilla S., Rivas G., Vicente M. (2012). Dynamic interaction of the escherichia coli cell division zipa and ftsz proteins evidenced in nanodiscs. J. Biol. Chem..

[14] Márquez I.F., Mateos-Gil P., Shin J.Y., Lagos R., Monasterio O., Vélez M. (2017). Mutations on ftsz lateral helix h3 that disrupt cell viability hamper reorganization of polymers on lipid surfaces. Biochim. Biophys. Acta Biomembr..

[15] Pichoff S., Lutkenhaus J. (2005). Tethering the z ring to the membrane through a conserved membrane targeting sequence in ftsa. Mol. Microbiol..

[16] Hale C.A., de Boer P.A.J. (1997). Direct binding of ftsz to zipa, an essential component of the septal ring structure that mediates cell division in *E. coli*. Cell.

[17] Ohashi T., Hale C.A., de Boer P.A.J., Erickson H.P. (2002). Structural evidence that the p/q domain of zipa is an unstructured, flexible tether between the membrane and the c-terminal ftsz-binding domain. J. Bacteriol..

[18] Buske P.J., Mittal A., Pappu R.V., Levin P.A. (2015). An intrinsically disordered linker plays a critical role in bacterial cell division. Semin. Cell Dev. Biol..

[19] Sundararajan K., Miguel A., Desmarais S.M., Meier E.L., Casey Huang K., Goley E.D. (2015). The bacterial tubulin ftsz requires its intrinsically disordered linker to direct robust cell wall construction. Nat. Commun..

[20] Sundararajan K., Goley E.D. (2017). The intrinsically disordered c-terminal linker of ftsz regulates protofilament dynamics and superstructure in vitro. J. Biol. Chem..

[21] Mateos-Gil P., Marquez I., Lopez-Navajas P., Jimenez M., Vicente M., Mingorance J., Rivas G., Velez M. (2012). Ftsz polymers bound to lipid bilayers through zipa form dynamic two dimensional networks. Biochim. Biophys. Acta.

[22] Encinar M., Kralicek A.V., Martos A., Krupka M., Cid S., Alonso A., Rico A.I., Jiménez M., Vélez M. (2013). Polymorphism of ftsz filaments on lipid surfaces: Role of monomer orientation. Langmuir.

[23] Loose M., Mitchison T.J. (2013). The bacterial cell division proteins ftsa and ftsz self-organize into dynamic cytoskeletal patterns. Nat. Cell Biol..

[24] Ramirez-Diaz D.A., García-Soriano D.A., Raso A., Mücksch J., Feingold M., Rivas G., Schwille P. (2018). Treadmilling analysis reveals new insights into dynamic ftsz ring architecture. PLoS Biol..

[25] Sundararajan K., Vecchiarelli A., Mizuuchi K., Goley E.D. (2018). Species- and c-terminal linker-dependent variations in the dynamic behavior of ftsz on membranes in vitro. Mol. Microbiol..

[26] Milam S.L., Osawa M., Erickson H. (2012). Negative-stain electron microscopy of inside-out ftsz rings reconstituted on artificial membrane tubules show ribbons of protofilaments. Biophys. J..

[27] Osawa M., Anderson D.E., Erickson H.P. (2008). Reconstitution of contractile ftsz rings in liposomes. Science.

[28] Osawa M., Anderson D.E., Erickson H.P. (2009). Curved ftsz protofilaments generate bending forces on liposome membranes. EMBO J..

[29] Gonzalez de Prado Salas P., Encinar M., Velez M., Tarazona P. (2013). Ftsz protein on bilayer membranes: Effects of specific lateral bonds. Soft Matter.

[30] Paez A., Mateos-Gil P., Hörger I., Mingorance J., Rivas G., Vicente M., Vélez M., Tarazona P. (2009). Simple modeling of ftsz polymers on flat and curved surfaces: Correlation with experimental in vitro observations. PMC Biophys..

[31] Hörger I., Velasco E., Mingorance J., Rivas G., Tarazona P., Vélez M. (2008). Langevin computer simulations of bacterial protein filaments and the force-generating mechanism during cell division. Phys. Rev. E.

[32] Hörger I., Velasco E., Rivas G., Vélez M., Tarazona P. (2008). Ftsz bacterial cytoskeletal polymers on curved surfaces: The importance of lateral interactions. Biophys. J..

[33] Gonzalez de Prado Salas P., Horger I., Martin-Garcia F., Mendieta J., Alonso A., Encinar M., Gomez-Puertas P., Velez M., Tarazona P. (2014). Torsion and curvature of ftsz filaments. Soft Matter.

[34] de Prado Salas P.G., Encinar M., Alonso A., Vélez M., Tarazona P. (2015). Modeling the interplay between protein and lipid aggregation in supported membranes. Chem. Phys. Lipids.

[35] González de Prado Salas P., Tarazona P. (2016). Collective effects of torsion in ftsz filaments. Phys. Rev. E.

[36] Arumugam S., Chwastek G., Fischer-Friedrich E., Ehrig C., Mönch I., Schwille P. (2012). Surface topology engineering of membranes for the mechanical investigation of the tubulin homologue ftsz. Angew. Chem. Int. Ed..

[37] Hsin J., Gopinathan A., Huang K.C. (2012). Nucleotide-dependent conformations of ftsz dimers and force generation observed through molecular dynamics simulations. Proc. Natl. Acad. Sci. USA.

[38] Qing-Ming Y., Lutkenhaus J. (1985). The nucleotide sequence of the essential cell-division gene ftsz of escherichia coli. Gene.

[39] Nogales E., Downing K.H., Amos L.A., Löwe J. (1998). Tubulin and ftsz form a distinct family of gtpases. Nat. Struct. Biol..

[40] Shahinian S., Silvius J.R. (1995). A novel strategy affords high-yield coupling of antibody fab´fragments to liposomes. Biochim. Biophys. Acta Biomembr..

[41] Huwyler J., Wu D., Pardridge W.M. (1996). Brain drug delivery of small molecules using immunoliposomes. Proc. Natl. Acad. Sci. USA.

[42] Mingorance J., Tadros M., Vicente M., Gonzalez J.M., Rivas G., Velez M. (2005). Visualization of single escherichia coli ftsz filament dynamics with atomic force microscopy. J. Biol.Chem..

[43] Chen Y., Erickson H.P. (2005). Rapid in vitro assembly dynamics and subunit turnover of ftsz demonstrated by fluorescence resonance energy transfer. J. Biol. Chem..

[44] Lu C., Stricker J., Erickson H.P. (2001). Site-specific mutations of ftsz - effects on gtpase and in vitro assembly. BMC Microbiol..

[45] Shin J.Y., Vollmer W., Lagos R., Monasterio O. (2013). Glutamate 83 and arginine 85 of helix h3 bend are key residues for ftsz polymerization, gtpase activity and cellular viability of escherichia coli: Lateral mutations affect ftsz polymerization and E. coli viability. BMC Microbiol..

[46] Jaiswal R., Patel R.Y., Asthana J., Jindal B., Balaji P.V., Panda D. (2010). E93r substitution of escherichia coli ftsz induces bundling of protofilaments, reduces gtpase activity, and impairs bacterial cytokinesis. J. Biol. Chem..

[47] Monahan L.G., Robinson A., Harry J.E. (2009). Lateral ftsz association and the assembly of the cytokinetic z ring in bacteria. Mol. Microbiol..

[48] Guan F., Yu J., Yu J., Liu Y., Li Y., Feng X.-H., Huang K.C., Chang Z., Ye S. (2018). Lateral interactions between protofilaments of the bacterial tubulin homolog ftsz are essential for cell division. eLife.

[49] Fontaine S.D., Reid R., Robinson L., Ashley G.W., Santi D.V. (2015). Long-term stabilization of maleimide–thiol conjugates. Bioconj. Chem..

[50] Watkins E.B., El-khouri R.J., Miller C.E., Seaby B.G., Majewski J., Marques C.M., Kuhl T.L. (2011). Structure and thermodynamics of lipid bilayers on polyethylene glycol cushions: Fact and fiction of peg cushioned membranes. Langmuir.

[51] Zhou X., Zhang Y., Zhang F., Pillai S., Liu J., Li R., Dai B., Li B., Zhang Y. (2013). Hierarchical ordering of amyloid fibrils on the mica surface. Nanoscale.

[52] Bisson-Filho A.W., Hsu Y.-P., Squyres G.R., Kuru E., Wu F., Jukes C., Sun Y., Dekker C., Holden S., VanNieuwenhze M.S. (2017). Treadmilling by ftsz filaments drives peptidoglycan synthesis and bacterial cell division. Science.

[53] Yang X., Lyu Z., Miguel A., McQuillen R., Huang K.C., Xiao J. (2017). Gtpase activity–coupled treadmilling of the bacterial tubulin ftsz organizes septal cell wall synthesis. Science.

[54] Torrance L., Ziegler A., Pittman H., Paterson M., Toth R., Eggleston I. (2006). Oriented immobilisation of engineered single-chain antibodies to develop biosensors for virus detection. J. Virol. Methods.

[55] Rivas G., López A., Mingorance J., Ferrándiz M.a.J., Zorrilla S., Minton A.P., Vicente M., Andreu J.M. (2000). Magnesium-induced linear self-association of the ftsz bacterial cell division protein monomer: The primary steps for ftsz assembly. J. Biol. Chem..

[56] Moreno-Herrero F., Pablo P.J.D., Fernández-Sánchez R., Colchero J., Gómez-Herrero J., Baró A.M. (2002). Scanning force microscopy jumping and tapping modes in liquids. Appl. Phys. Lett..

