# Supplementary material for: Surface Orientation and Binding Strength Modulate Shape of FtsZ on Lipid Surfaces
Source: Int J Mol Sci. 2019 May 24;20(10):2545. doi: 10.3390/ijms20102545 (PMC6566678; doi:10.3390/ijms20102545)
Supplement: Supplementary file 1 [file ijms-20-02545-s001.zip › SI.pdf]

SUPLEMENTARY INFORMATION

Surface orientation and binding strength modulate shape of FtsZ on lipid surfaces

Ileana Márquez, Gabriel Díaz-Haro, Marisela Vélez*

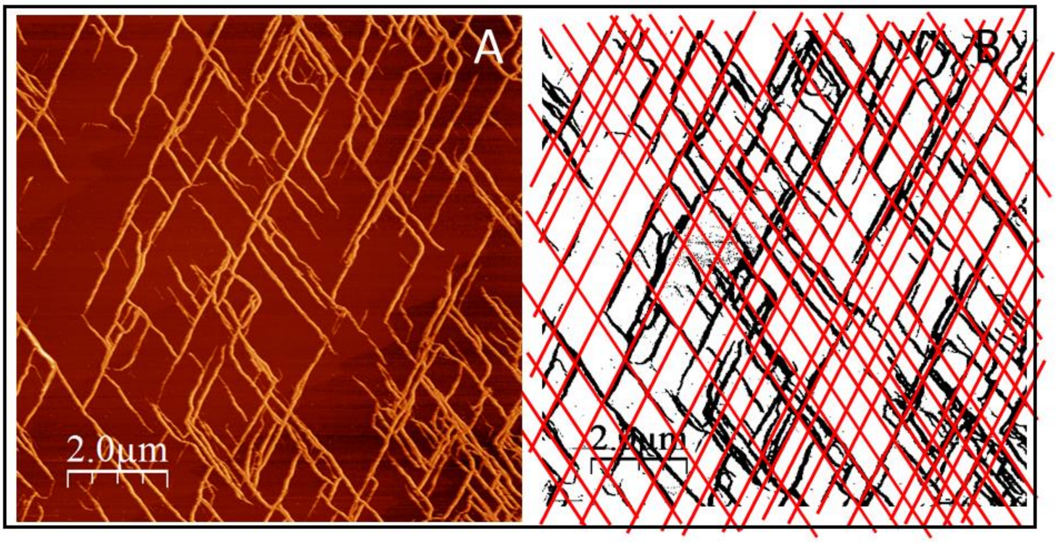

S1 Panel A shows filaments grown on the bilayer. At the micrometer range the filaments are aligned preferentially at 60 degree angles. Panel B show the filaments under a superimposed reticle of lines oriented at 60 degrees.

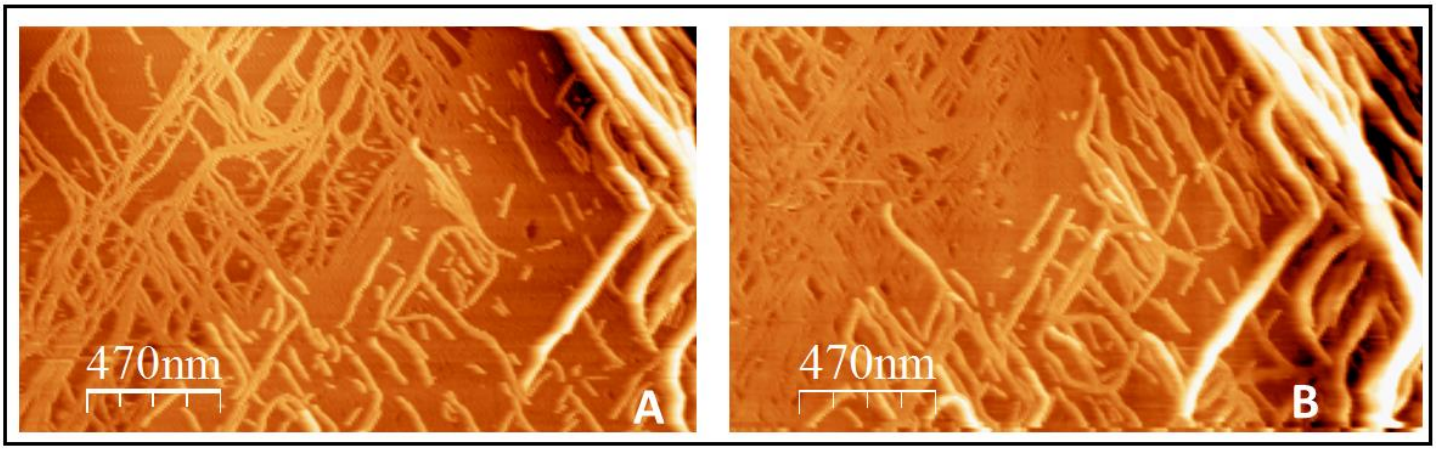

S2 Image B was taken 11 minutes after A. The formation of a dense layer of filaments followed by increase growth in three dimensions of a second and third filament layer is observed, before the formation of thicker less defined bundles.

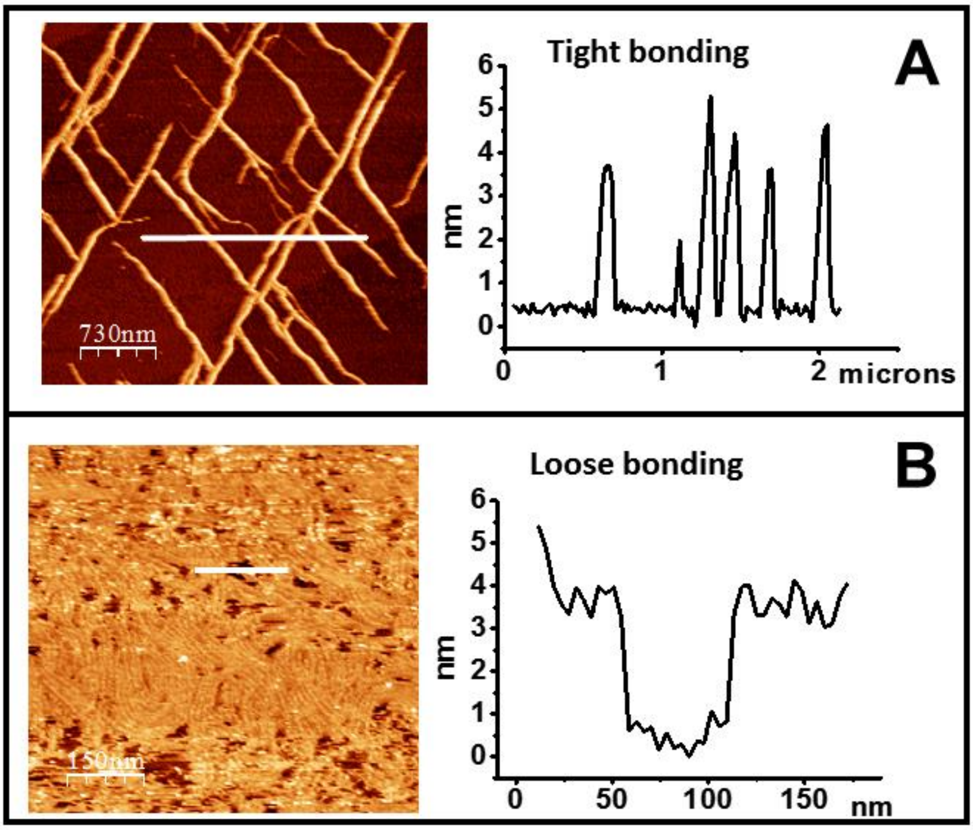

S3 Height of the observed filaments under the two attachments. The observed 4 nm correspond to the height expected for individual filaments.
